# IDO Inhibition by 1-Methyltryptophan: Unlocking New Paths to Treat Ovarian Dysfunction and Hormonal Imbalance in PCOS

**DOI:** 10.5812/ijpr-164861

**Published:** 2025-09-06

**Authors:** Hediyeh Gheymoumi, Sevda Shayesteh, Marziyeh Amiri-Andebilib, Naim Gheymoumi, Fatemeh Sharifi, Ahmad Reza Dehpour

**Affiliations:** 1 Department of Pharmacology and Toxicology, Faculty of Pharmacy, Alborz University of Medical Sciences, Karaj, Iran; 2 Student Research Committee, Alborz University of Medical Sciences, Karaj, Iran; 3 Department of Pharmacology, School of Medicine, Tehran University of Medical Sciences, Tehran, Iran; 4 Experimental Medicine Research Center, Tehran University of Medical Sciences, Tehran, Iran

**Keywords:** Polycystic Ovary Syndrome, Indoleamine 2,3-Dioxygenase, 1-Methyltryptophan, IRS-1 Protein, Insulin

## Abstract

**Background:**

Polycystic ovary syndrome (PCOS) is a common endocrine disorder characterized by chronic inflammation, as well as metabolic and reproductive dysfunction. While insulin resistance affects many tissues, ovarian tissue exhibits insulin hypersensitivity, which promotes androgen excess and worsens PCOS symptoms. The kynurenine pathway (KP), a major route of tryptophan metabolism regulated by indoleamine 2,3-dioxygenase (IDO), is implicated in ovarian dysfunction in PCOS.

**Objectives:**

To investigate the effects of IDO inhibition on ovarian morphology and insulin signaling in a PCOS rat model, evaluating its potential as a therapeutic approach compared to metformin.

**Methods:**

Twenty-four female rats were randomly assigned to four groups: Control, PCOS, 1-methyltryptophan (1-MT, IDO inhibitor) (10 mg/kg), and metformin (100 mg/kg). The PCOS was induced by subcutaneous testosterone injections at 21 days of age. Outcomes measured included luteinizing hormone/follicle-stimulating hormone (LH/FSH) ratio, ovarian weight, follicle and corpus luteum counts, granulosa and theca layer thickness, fasting blood glucose, and ovarian expression of insulin receptor substrate 1 (IRS-1) and phosphoinositide 3-kinase (PI3K).

**Results:**

The results indicated that both treatments, 1-MT and metformin, significantly reduced the LH/FSH ratio, with metformin showing a more substantial effect. Additionally, 1-MT increased ovarian weight and the number of healthy follicles (HFs), unlike metformin. Both treatments increased the number of corpora lutea (CL), indicating restored ovulation. The IRS-1 expression decreased with both treatments, whereas PI3K levels remained unchanged.

**Conclusions:**

The IDO inhibition by 1-MT improves ovarian function and hormonal balance in PCOS more effectively than metformin, likely by reducing inflammation and modulating insulin signaling. These findings support 1-MT as a promising therapeutic candidate for PCOS management and improving ovarian function. However, these results are from a short-term animal study, and further clinical trials are necessary to assess long-term efficacy and safety.

## 1. Background

Polycystic ovary syndrome (PCOS) is a prevalent endocrine-metabolic disorder affecting women of reproductive age, characterized by hyperandrogenism, ovarian dysfunction, and metabolic disturbances such as insulin resistance (1-3). Insulin resistance, present in 50% to 90% of PCOS patients, significantly contributes to its pathogenesis (4). Despite systemic insulin resistance, ovarian tissue in PCOS paradoxically exhibits insulin hypersensitivity, enhancing androgen biosynthesis in theca cells synergistically with luteinizing hormone (LH) (5, 6). This process involves insulin receptor activation, leading to the phosphorylation of insulin receptor substrates (IRS), particularly insulin receptor substrate 1 (IRS-1), and subsequent phosphoinositide 3-kinase (PI3K)/Akt signaling, which increases 17α-hydroxylase activity and androgen production. The PI3K inhibitors can block this effect, underscoring the pathway’s role in PCOS-related steroidogenesis (7). Additionally, insulin can disrupt ovulation by increasing aromatase activity in granulosa cells and amplifying LH action (8, 9). Lifestyle modifications and insulin-sensitizing agents like metformin are standard treatments for PCOS due to the central role of insulin resistance (10). While metformin improves ovulatory function and insulin sensitivity, its gastrointestinal side effects and the rare risk of lactic acidosis limit long-term use, prompting interest in novel therapies (11, 12).

Recent evidence highlights the kynurenine pathway (KP) of tryptophan metabolism as a contributor to PCOS pathogenesis (13). Indoleamine 2,3-dioxygenase (IDO), the rate-limiting enzyme in this pathway, is upregulated by pro-inflammatory cytokines, leading to tryptophan depletion and kynurenine metabolite accumulation (14). The IDO activity impacts immune tolerance and metabolic processes, impairing insulin sensitivity in disease models (15). Kynurenines also modulate immune responses, linking inflammation and metabolism (16). In PCOS, elevated IDO activity is associated with chronic inflammation (17), with clinical studies reporting increased kynurenine levels correlating with inflammatory markers and oxidative stress (18, 19). This IDO overactivity may exacerbate inflammation and metabolic dysfunction rather than compensate.

## 2. Objectives

This study investigates the effects of IDO inhibition in a PCOS rat model, focusing on ovarian function, insulin signaling, and hormonal regulation, aiming to clarify the therapeutic potential of targeting this pathway.

## 3. Methods

### 3.1. Animals

All procedures adhered to the animal care standards of Alborz University of Medical Sciences (IR.ABZUMS.AEC.1402.007). Rats were housed at 25°C, 40% humidity, with a 12-hour light/dark cycle and unrestricted food and water access. Twenty-four female Wistar rats were randomly assigned to four groups (six per group): Control (saline injection), PCOS, 1-methyltryptophan (1-MT), and metformin. The rats were 7 days old at the start of the study, with an initial average weight of 15 - 18 g. At the end of the PCOS induction period (day 35), the average weight of the animals was approximately 75 - 80 g. A block randomization method was used to ensure an equal distribution of rats across the four experimental groups. Due to the nature of the interventions (e.g., intraperitoneal injection vs. oral gavage), blinding of the personnel administering the treatments was not possible. However, the researcher performing the hormonal and histopathological analyses was blinded to the group assignments to minimize potential bias.

### 3.2. Polycystic Ovary Syndrome and Treatment Procedure

The PCOS was induced using subcutaneous testosterone injections (1 mg/100 g BW) on days 7, 14, 21, 28, and 35 post-birth (20). To simulate the injection procedure, the Control group received normal saline injections at the same dosage and time intervals. Stable follicle-stimulating hormone (FSH), LH, and testosterone levels confirmed the absence of PCOS. Blood samples were collected from the tail vein. From day 35, the 1-MT group received 1-MT (10 mg/kg; Sigma Aldrich, Germany) via intraperitoneal injection for 14 consecutive days (21). Concurrently, the metformin group received metformin (100 mg/kg; AryaPharm, Iran) via oral gavage for the same duration (22). Following the treatment period, all rats were euthanized via cardiac dissection while under anesthesia induced by a mixture of ketamine (60 mg/kg; Alfasan, Netherlands) and xylazine (10 mg/kg; Serumwerk, Germany), both administered intraperitoneally. Blood samples were collected, centrifuged, and the resulting serum was stored at -80°C for future analysis.

### 3.3. Blood Sugar Measurement

Fasting blood sugar was measured from the tail vein using a glucometer (Accu-Chek Performa) on the first and last experiment days to ensure no baseline differences between groups. To obtain these measurements, rats were fasted for 12 hours before blood collection, while having unrestricted access to water. This procedure was performed to ensure accurate fasting blood glucose readings and to confirm there were no baseline differences between the groups.

### 3.4. Hormonal and Biomolecular Tests

The LH/FSH ratio, a key hormonal marker of hypothalamic-pituitary-ovarian (HPO) axis disruptions in PCOS, was quantified using rat-specific enzyme-linked immunosorbent assay (ELISA) kits. The LH and FSH levels were measured with kits BS764675 and BS2021901, respectively (MyBioSource, USA). To investigate molecular alterations in insulin signaling pathways, IRS-1 and PI3K protein levels in ovarian tissue homogenates were measured using specific ELISA kits (IRS-1: MBS029127; PI3K: MBS9518759, MyBioSource, USA). Tissue samples were collected post-euthanasia, weighed, minced, and homogenized in PBS (10 mg tissue per 100 µL). After centrifugation at 1000 × g for 20 minutes, the supernatant was used for protein analysis. Following the ELISA kit protocols, samples were added to plates, incubated, washed, and exposed to detection solution. Absorbance was measured at 450 nm to quantify ovarian IRS-1, PI3K, and serum LH and FSH levels.

### 3.5. Histopathology

To control for the stage of the estrous cycle at the time of tissue collection, vaginal smears were performed on each animal for 10 consecutive days, starting from day 43 after birth. From day 49 (the final day of treatment) to day 53, each animal was monitored daily. Once an animal entered the metestrus/diestrus phase, it was euthanized, and ovarian tissue was collected immediately thereafter. The animals were still in the process of achieving full puberty, and estrous cycle irregularities were possible, making this detailed monitoring necessary.

All smears were collected and recorded daily between 08:00 and 10:00 AM. Euthanasia and tissue collection were then synchronized to occur when each animal was in the metestrus/diestrus phase. This specific phase was chosen because it lacks the LH/estradiol surge of proestrus, providing a more stable hormonal and tissue profile for consistent histological and molecular analysis. This procedure ensured that all tissue collection was standardized, minimizing hormonal variability as a confounding factor. Ovarian tissues were excised, fixed in 10% neutral buffered formalin for 48 hours, then dehydrated, cleared, and paraffin-embedded. Six µm serial sections were obtained via microtome and H&E stained for histological evaluation. Three non-consecutive tissue sections were randomly selected by an individual blinded to the experimental groups. This protocol allowed assessment of ovarian structural integrity and functional state post-PCOS induction and treatment by evaluating ovarian weight, healthy and atretic follicle counts, granulosa and theca layer thickness, and corpus luteum presence (indicating ovulatory activity).

For histopathological comparison, healthy follicles (HFs) showed organized granulosa cells with intact nuclei, intact oocytes with clear cytoplasm, well-defined fluid-filled antra, continuous basement membranes, normal theca layers, and regular shapes, lacking cell death markers. Atretic follicles, conversely, displayed disorganized granulosa cells with nuclear pyknosis, degenerated oocytes, collapsed antra, disrupted basement membranes, irregular theca layers, shrunken shapes, and signs of apoptosis/necrosis. These parameters were assessed under light microscopy.

### 3.6. Data Analysis

Statistical analysis was performed using GraphPad Prism version 8 software. The sample size for each group was six animals (n = 6). Group differences were analyzed via one-way ANOVA, with Tukey’s post-hoc test for multiple comparisons. A P-value < 0.05 was considered statistically significant.

## 4. Results

### 4.1. Blood Sugar Measurement

**Figure 1.**
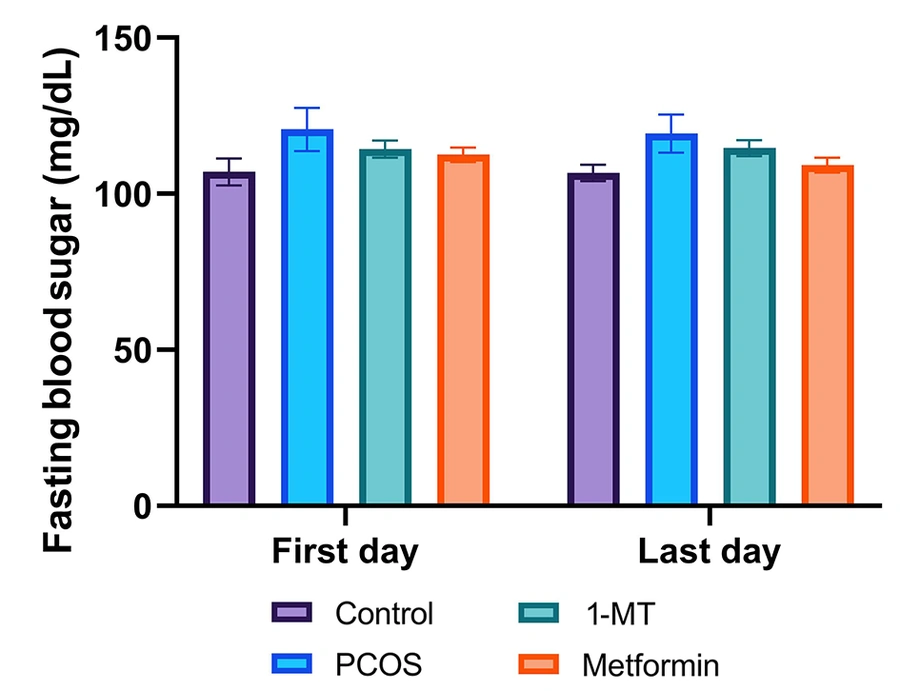
Fasting blood sugar levels across experimental groups at the beginning and end of the study. There was no significant difference in blood glucose levels among any of the groups at baseline (first day) or after the treatment period (last day; P = 0.69, one-way ANOVA). Data are presented as mean ± SEM (n = 6).

There was no significant difference in fasting blood sugar among groups (P = 0.69; Figure 1). 

### 4.2. Hormonal Changes

**Figure 2.**
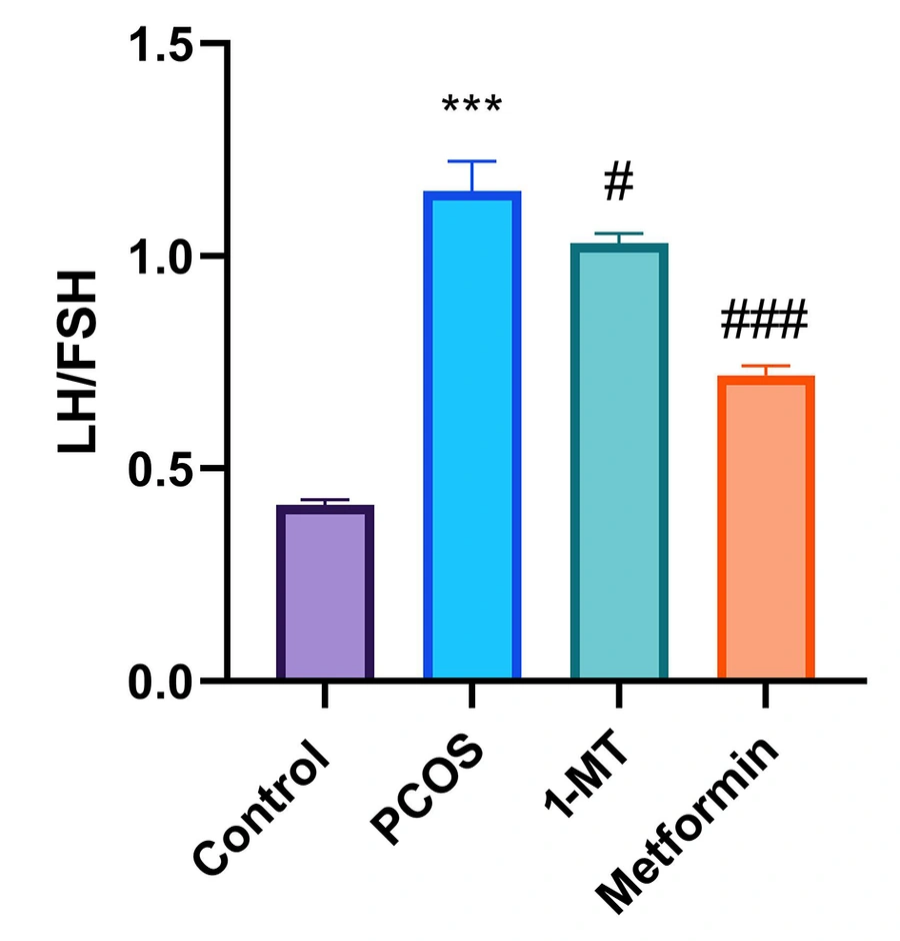
Luteinizing hormone/follicle-stimulating hormone (LH/FSH) ratio comparison among control, polycystic ovary syndrome (PCOS), and treated groups (significant differences: *** P < 0.001 vs. control; # P < 0.05, ### P < 0.001 vs. PCOS; data are presented as mean ± SEM; n = 6).

The LH/FSH ratio in the PCOS group significantly increased compared to the control group (P < 0.0001). Both 1-MT and metformin reduced this ratio compared to PCOS (P = 0.032, P < 0.0001), with metformin showing a more substantial effect (P < 0.0001; Figure 2). 

### 4.3. Ovarian Status

**Figure 3.**
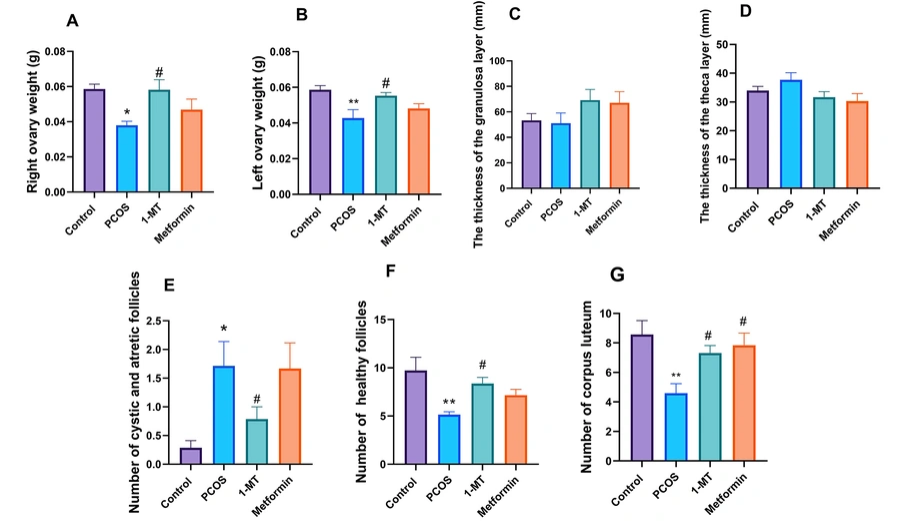
Ovarian tissue analysis: Weights of right (A) and left (B) ovaries; thickness of granulosa (C) and theca (D) layers. Follicle assessment in ovaries: (E) atretic and cystic follicles (CFs), (F) healthy follicles (HFs), and (G) corpus luteum counts [statistical significance: * P < 0.01, ** P < 0.01 vs. control, # P < 0.05 vs. polycystic ovary syndrome (PCOS); data are presented as mean ± SEM; n = 6].

Ovarian weight significantly decreased in the PCOS group compared to controls (Figure 3A, P = 0.032; Figure 3B, P = 0.004). Treatment with 1-MT restored ovarian weight (Figure 3A, P = 0.037; Figure 3B, P = 0.034), while metformin did not significantly affect ovarian weight (Figure 3A, P = 0.57; Figure 3B, P = 0.58). Granulosa and theca layer thickness showed no significant changes across groups (Figure 3C, P = 0.29; Figure 3D, P = 0.096).

### 4.4. Follicular Status

The PCOS group showed increased atretic and cystic follicles (CFs) compared to controls (P = 0.013). The 1-MT significantly reduced CFs and increased HFs compared to PCOS (Figure 3E, P = 0.018; Figure 3F, P = 0.044), while metformin had no significant effect (Figure 3E, P = 0.99; Figure 3F, P = 0.34). Corpus luteum counts also increased with both treatments compared to the PCOS group, indicating improved ovulation (Figure 3G, P = 0.029; Figure 3G, P = 0.026).

**Figure 4.**
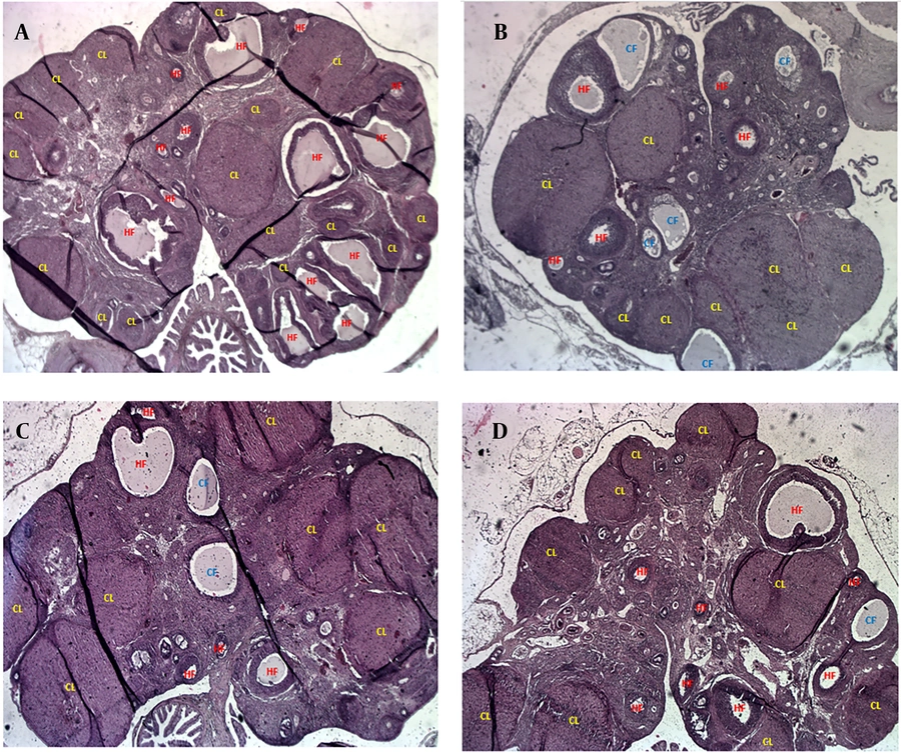
Representative histological images showing healthy (HFs), cystic follicles (CFs), and corpus luteum (CL) in ovarian tissue (magnification ×40): A, control; B, polycystic ovary syndrome (PCOS); C, 1-methyltryptophan (1-MT); D, metformin.

Figure 4 presents representative H&E-stained ovarian tissue images illustrating the impact of PCOS and treatments. Figure 4A (control) shows healthy ovarian morphology with numerous HFs and abundant corpora lutea (CL), indicative of normal ovulatory cycles. In contrast, Figure 4B (PCOS) reveals significant dysfunction, characterized by an increase in CFs displaying signs of atresia (disorganized granulosa cells, degenerated oocytes, collapsed antra) and reduced HFs and CL. Figure 4C (1-MT) demonstrates significant morphological improvements, with a marked increase in HFs and numerous well-formed CL, alongside a reduction in CFs, reflecting restored ovarian function. Figure 4D (metformin) also shows improved ovarian morphology compared to PCOS, particularly an increase in CL, suggesting restored ovulation. However, the overall follicular architecture and reduction in CFs are less pronounced in the metformin group compared to the 1-MT group. These visual findings consistently support the quantitative results, highlighting 1-MT's superior impact on ovarian remodeling and follicular health.

### 4.5. Molecular Results

**Figure 5.**
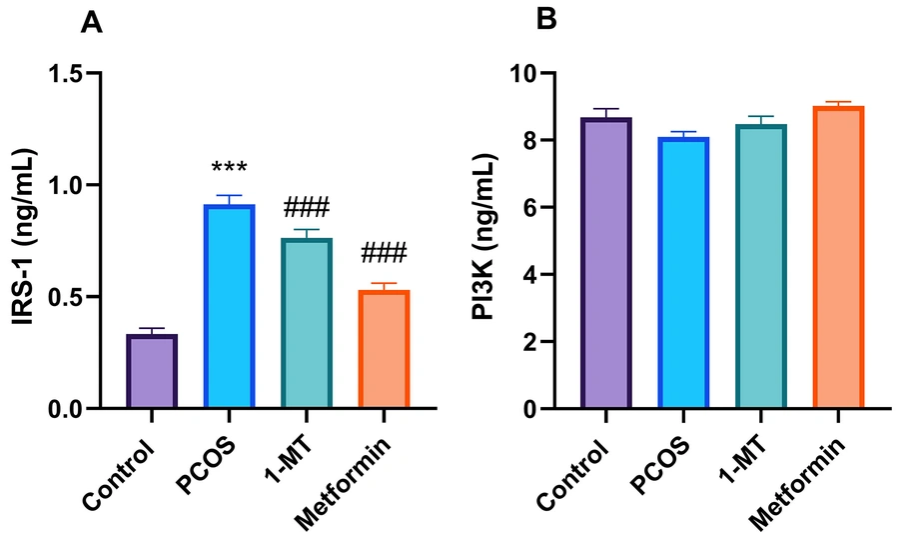
Ovarian expression of insulin signaling markers: A, insulin receptor substrate 1 (IRS-1) protein levels with significant changes [*** P < 0.001 vs. control; ### P < 0.001 vs. polycystic ovary syndrome (PCOS)]; B, phosphoinositide 3-kinase (PI3K) protein levels across groups (data are presented as mean ± SEM; n = 6).

The IRS-1 protein levels significantly increased in the PCOS group compared to controls (P < 0.0001). Both 1-MT and metformin reduced IRS-1 protein levels (P < 0.0001), while PI3K protein expression showed no significant changes (P = 0.28; Figure 5). 

## 5. Discussion

The IDO is a key immunoregulatory enzyme whose activity is upregulated by pro-inflammatory cytokines such as interferon gamma (IFN-γ), tumor necrosis factor alpha (TNF-α), and interleukin-6 (IL-6), all of which are elevated in PCOS (23-25). This upregulation drives the KP of tryptophan metabolism, leading to the accumulation of metabolites that sustain inflammation, promote immune tolerance, and contribute to metabolic dysfunction (14, 17). Our study utilized 1-MT, an IDO inhibitor, to interrupt this inflammatory cycle and investigate its therapeutic potential compared to metformin, a standard PCOS treatment. This approach offers a novel therapeutic strategy that targets an upstream driver of PCOS pathogenesis, moving beyond traditional insulin-sensitizing mechanisms.

The PCOS is characterized by a disrupted HPO axis, which manifests as an elevated LH/FSH ratio (26). Both 1-MT and metformin treatments significantly reduced this ratio compared to the PCOS group, indicating a partial restoration of HPO axis feedback. However, our data show that metformin had a more pronounced effect on normalizing this ratio. This finding is consistent with metformin's well-established direct influence on improving systemic insulin sensitivity and modulating the central regulation of gonadotropin-releasing hormone (GnRH) pulse frequency (11). In contrast, 1-MT’s positive effect on the LH/FSH ratio is likely an indirect consequence of its anti-inflammatory effects. By suppressing inflammatory cytokines that can disrupt neuroendocrine signaling, 1-MT appears to contribute to a more balanced hormonal milieu, consistent with previous findings linking inflammation to HPO axis dysfunction (23, 27, 28).

Our findings on ovarian morphology provide a crucial distinction between the two treatments. While both 1-MT and metformin increased the number of CL, which is a key indicator of restored ovulation, only 1-MT significantly increased ovarian weight and the number of HFs. This highlights 1-MT’s superior efficacy in promoting ovarian remodeling and overall follicular health. The observation that ovulation was restored despite unchanged granulosa and theca layer thickness requires further clarification. This apparent inconsistency may be explained by the short, 14-day treatment period. This duration was likely sufficient to trigger the final maturation and ovulation of existing, advanced-stage follicles but was too brief to induce a statistically significant proliferation and increase in the overall thickness of these layers across the entire cohort of developing follicles. It is also plausible that the treatments primarily promoted the proper differentiation and function of granulosa and theca cells, enabling successful ovulation without a substantial increase in cell numbers.

Regarding insulin signaling, our results show that both 1-MT and metformin significantly reduced ovarian IRS-1 protein levels in the PCOS group. This is a key finding, as ovarian insulin hypersensitivity, mediated by IRS-1, is a hallmark of PCOS-related androgen excess (29-31). However, PI3K protein levels remained unchanged across all groups. This observation is interesting and can be explained by several mechanisms. First, while IRS-1 is a key upstream activator of PI3K, the total abundance of PI3K protein may not be the primary regulatory point for its function. The functional output of the pathway is often regulated by phosphorylation (activation) of PI3K, not just its concentration. Second, other IRS isoforms, such as IRS-2, may have compensated for the reduction in IRS-1, maintaining PI3K activity. Third, both 1-MT and metformin may exert their beneficial effects on insulin signaling through other pathways. For instance, metformin is known to activate AMPK, which can inhibit mTOR and reduce inhibitory phosphorylation of IRS-1, making the remaining IRS-1 more functionally efficient. Similarly, 1-MT may modulate other IRS-1-mediated pathways, such as the MAPK/ERK cascade, which contributes to steroidogenesis and cell proliferation independently of PI3K protein levels (7, 32, 33).

In summary, the KP is a critical link between immune dysregulation, insulin signaling, and ovarian dysfunction in PCOS. Our findings suggest that 1-MT likely acts by suppressing cytokine-induced tryptophan catabolism, thereby creating a less inflammatory ovarian microenvironment that facilitates follicular maturation and ovulation. While metformin's primary mechanism is metabolic, acting via AMPK to improve insulin sensitivity, 1-MT provides a novel, inflammation-targeted strategy that addresses upstream causes of endocrine and metabolic dysfunction. We acknowledge that the therapeutic use of 1-MT, while promising, requires a thorough evaluation of its safety profile and long-term effects through future preclinical and clinical studies. Our results underscore the distinct and superior effects of IDO inhibition on ovarian health and function, highlighting its therapeutic promise in PCOS.

### 5.1. Conclusions

This study shows that 1-MT improves ovarian function, hormone balance, and follicular health more effectively than metformin. While metformin targets insulin resistance, 1-MT’s impact on ovarian morphology is stronger, likely due to its anti-inflammatory effects via the KP. These findings highlight 1-MT as a promising PCOS therapy.

### 5.2. Limitations

The main limitation of this study is the use of an animal model, which may not fully reflect human PCOS. The short treatment period also limits understanding of long-term effects.

### 5.3. Future Directions

Future studies should include clinical trials to assess 1-MT’s efficacy, safety, dose-response effect, and long-term impact, along with its molecular effects on inflammatory pathways.

## Data Availability

The dataset presented in this study is available upon request from the corresponding author, either during submission or after publication. The data are not publicly available due to central regulatory requirements governing the research.

## References

[1] Azziz R, Carmina E, Dewailly D, Diamanti-Kandarakis E, Escobar-Morreale HF, Futterweit W (2009). The Androgen Excess and PCOS Society criteria for the polycystic ovary syndrome: the complete task force report.. Fertil Steril..

[2] Luo Y, Cui C, Han X, Wang Q, Zhang C (2021). The role of miRNAs in polycystic ovary syndrome with insulin resistance.. J Assist Reprod Genet..

[3] Salimi-Asl M, Mozdarani H, Kadivar M (2016). Up-regulation of miR-21 and 146a expression and increased DNA damage frequency in a mouse model of polycystic ovary syndrome (PCOS).. Bioimpacts..

[4] Venkatesan AM, Dunaif A, Corbould A (2001). Insulin resistance in polycystic ovary syndrome: progress and paradoxes.. Recent Prog Horm Res..

[5] Stepto NK, Moreno-Asso A, McIlvenna LC, Walters KA, Rodgers RJ (2019). Molecular Mechanisms of Insulin Resistance in Polycystic Ovary Syndrome: Unraveling the Conundrum in Skeletal Muscle?. J Clin Endocrinol Metab..

[6] Murayama C, Miyazaki H, Miyamoto A, Shimizu T (2012). Luteinizing hormone (LH) regulates production of androstenedione and progesterone via control of histone acetylation of StAR and CYP17 promoters in ovarian theca cells.. Mol Cell Endocrinol..

[7] Munir I, Yen HW, Geller DH, Torbati D, Bierden RM, Weitsman SR (2004). Insulin augmentation of 17alpha-hydroxylase activity is mediated by phosphatidyl inositol 3-kinase but not extracellular signal-regulated kinase-1/2 in human ovarian theca cells.. Endocrinology..

[8] Mayer SB, Evans WS, Nestler JE (2015). Polycystic ovary syndrome and insulin: our understanding in the past, present and future.. Womens Health (Lond)..

[9] Pierre A, Peigne M, Grynberg M, Arouche N, Taieb J, Hesters L (2013). Loss of LH-induced down-regulation of anti-Mullerian hormone receptor expression may contribute to anovulation in women with polycystic ovary syndrome.. Hum Reprod..

[10] Wild RA, Carmina E, Diamanti-Kandarakis E, Dokras A, Escobar-Morreale HF, Futterweit W (2010). Assessment of cardiovascular risk and prevention of cardiovascular disease in women with the polycystic ovary syndrome: a consensus statement by the Androgen Excess and Polycystic Ovary Syndrome (AE-PCOS) Society.. J Clin Endocrinol Metab..

[11] Naderpoor N, Shorakae S, de Courten B, Misso ML, Moran LJ, Teede HJ (2015). Metformin and lifestyle modification in polycystic ovary syndrome: systematic review and meta-analysis.. Hum Reprod Update..

[12] Wang GS, Hoyte C (2019). Review of Biguanide (Metformin) Toxicity.. J Intensive Care Med..

[13] Davis I, Liu A (2015). What is the tryptophan kynurenine pathway and why is it important to neurotherapeutics?. Expert Rev Neurother..

[14] Wang Q, Liu D, Song P, Zou MH (2015). Tryptophan-kynurenine pathway is dysregulated in inflammation, and immune activation.. Front Biosci (Landmark Ed)..

[15] Prendergast GC, Metz R, Muller AJ, Merlo LM, Mandik-Nayak L (2014). IDO2 in Immunomodulation and Autoimmune Disease.. Frontiers Immunol..

[16] Cully M (2018). Metabolic disorders: IDO inhibitors could change tack to treat metabolic disorders.. Nat Rev Drug Discov..

[17] Jovanovic F, Sudhakar A, Knezevic NN (2022). The Kynurenine Pathway and Polycystic Ovary Syndrome: Inflammation as a Common Denominator.. Int J Tryptophan Res..

[18] Mancini A, Bruno C, Vergani E, d'Abate C, Giacchi E, Silvestrini A (2021). Oxidative Stress and Low-Grade Inflammation in Polycystic Ovary Syndrome: Controversies and New Insights.. Int J Mol Sci..

[19] Wang S, Mu L, Zhang C, Long X, Zhang Y, Li R (2022). Abnormal Activation of Tryptophan-Kynurenine Pathway in Women With Polycystic Ovary Syndrome.. Front Endocrinol (Lausanne)..

[20] Shirooie S, Khaledi E, Dehpour AR, Noori T, Khazaei M, Sadeghi F (2021). The effect of dapsone in testosterone enanthate-induced polycystic ovary syndrome in rat.. J Steroid Biochem Mol Biol..

[21] Shayesteh S, Guillemin GJ, Rashidian A, Faghir-Ghanesefat H, Mani AR, Tavangar SM (2021). 1-Methyl tryptophan, an indoleamine 2,3-dioxygenase inhibitor, attenuates cardiac and hepatic dysfunction in rats with biliary cirrhosis.. Eur J Pharmacol..

[22] Sander V, Luchetti CG, Solano ME, Elia E, Di Girolamo G, Gonzalez C (2006). Role of the N, N'-dimethylbiguanide metformin in the treatment of female prepuberal BALB/c mice hyperandrogenized with dehydroepiandrosterone.. Reproduction..

[23] Mohammadi S, Kayedpoor P, Karimzadeh-Bardei L, Nabiuni M (2017). The Effect of Curcumin on TNF-alpha, IL-6 and CRP Expression in a Model of Polycystic Ovary Syndrome as an Inflammation State.. J Reprod Infertil..

[24] Rudnicka E, Suchta K, Grymowicz M, Calik-Ksepka A, Smolarczyk K, Duszewska AM (2021). Chronic Low Grade Inflammation in Pathogenesis of PCOS.. Int J Mol Sci..

[25] Stone TW, Williams RO (2023). Interactions of IDO and the Kynurenine Pathway with Cell Transduction Systems and Metabolism at the Inflammation-Cancer Interface.. Cancers (Basel)..

[26] Pratama G, Wiweko B, Widyahening IS, Andraini T, Bayuaji H, Asmarinah (2024). Mechanism of elevated LH/FSH ratio in lean PCOS revisited: a path analysis.. Sci Rep..

[27] Zhang Y, Shi H, Yang G, Yang Y, Li W, Song M (2021). Associations between expression of indoleamine 2, 3-dioxygenase enzyme and inflammatory cytokines in patients with first-episode drug-naive Schizophrenia.. Transl Psychiatry..

[28] Tiwari S, Paramanik V (2025). Role of Probiotics in Depression: Connecting Dots of Gut-Brain-Axis Through Hypothalamic-Pituitary Adrenal Axis and Tryptophan/Kynurenic Pathway involving Indoleamine-2,3-dioxygenase.. Mol Neurobiol..

[29] Villuendas G, Botella-Carretero JI, Roldan B, Sancho J, Escobar-Morreale HF, San Millan JL (2005). Polymorphisms in the insulin receptor substrate-1 (IRS-1) gene and the insulin receptor substrate-2 (IRS-2) gene influence glucose homeostasis and body mass index in women with polycystic ovary syndrome and non-hyperandrogenic controls.. Hum Reprod..

[30] Wu X, Sallinen K, Anttila L, Makinen M, Luo C, Pollanen P (2000). Expression of insulin-receptor substrate-1 and -2 in ovaries from women with insulin resistance and from controls.. Fertil Steril..

[31] Wu XK, Zhou SY, Liu JX, Pollanen P, Sallinen K, Makinen M (2003). Selective ovary resistance to insulin signaling in women with polycystic ovary syndrome.. Fertil Steril..

[32] Li T, Mo H, Chen W, Li L, Xiao Y, Zhang J (2017). Role of the PI3K-Akt Signaling Pathway in the Pathogenesis of Polycystic Ovary Syndrome.. Reprod Sci..

[33] Banerjee J, Bruckbauer A, Zemel MB (2016). Activation of the AMPK/Sirt1 pathway by a leucine-metformin combination increases insulin sensitivity in skeletal muscle, and stimulates glucose and lipid metabolism and increases life span in Caenorhabditis elegans.. Metabolism..

